# Exploring the applicability of a multifactor mindfulness scale in the Chinese college context

**DOI:** 10.3389/fpsyg.2024.1415692

**Published:** 2024-07-02

**Authors:** Dan Zhang, Jianbo Shen, Hongyu Ma

**Affiliations:** ^1^School of Psychology, Central China Normal University (CCNU), Wuhan, China; ^2^Zhixing College of Hubei University, Wuhan, China; ^3^The National Tax Institute of STA, Yangzhou, China

**Keywords:** mindfulness, the Chinese comprehensive inventory of mindfulness experiences, factor structure validation, reliability, validity

## Abstract

The development of a precise and comprehensive mindfulness measurement tool is a compelling area of research due to its lack at present. This study examines the utility of a multifactor mindfulness scale, particularly the Chinese version of the Comprehensive Inventory of Mindfulness Experiences (CHIME), among Chinese college students. Prior to formal testing, 410 subjects completed the CHIME-37, providing feedback for refinement. During formal assessment, 1,785 subjects participated, with 490 students retested after 2 months. The validity of the CHIME-37 was evaluated using various scales, including subjective well-being, psychological well-being, peace of mind, self-reflection, insight, emotion regulation, depression-anxiety-stress, and sickness questionnaire. In exploratory factor analysis of Sample 1 (*n* = 838), CHIME revealed 8 factors, explaining 70.696% of the variance. Confirmatory factor analysis in Sample 2 (*n* = 947) confirmed the 8-factor model’s validity. Internal consistency coefficients ranged from 0.848 to 0.914, with test–retest reliabilities ranging from 0.746 to 0.885, and split-half reliabilities ranging from 0.795 to 0.898. Total and dimension scores correlated positively with subjective well-being, psychological well-being, emotion stability, and cognitive reappraisal (*p* < 0.01) but negatively with physical and mental illnesses, depression-anxiety-stress, and expressive inhibition (*p* < 0.01). The revised CHIME demonstrates robust reliability and validity, establishing it as a suitable tool for measuring the mindfulness levels of Chinese college students.

## 1 Introduction

At present, over 10 tools are available for assessing mindfulness; however, none of them can accurately capture all attributes of mindfulness. In addition, a consensus on the specific traits encompassed within mindfulness is lacking to date (Malinowski, 2008). Currently, the widely accepted definition of mindfulness is the one given by Kabat-Zinn who defined it is as “A purposeful, nonjudgmental attention to present moment awareness (Kabat-Zinn, 2003).” Mindfulness is viewed as a practice that involves fostering a curious, open, nonjudgmental, and accepting attitude. It directs attention and awareness to internal and external stimuli in the present moment, encompassing emotions, cognition, and bodily sensations like touch, taste, smell, and breath (Gu et al., 2015). Different constructs and understandings of mindfulness have led to different definitions of mindfulness: Experience-oriented mindfulness views mindfulness as an individual’s awareness of various present moment mind–body experiences, thus placing emphasis on awareness and acceptance (Peng and Ju, 2013). The proficiency-oriented approach encompasses a range of mindfulness practices, namely mindfulness meditation, mindfulness attention training, and purely mental mindfulness exercises (Baer et al., 2004). The competence-oriented perspective regards mindfulness as an individual’s inherent capacity, suggesting that mindfulness abilities or skills can be enhanced through mindfulness practices or exercises such as mindful breathing and walking. The trait-oriented approach perceives mindfulness as a trait-like variable, comparable to character strengths or virtues in positive psychology. Mindfulness, influenced by genetic and environmental factors, is a unique individual difference factor and a personality trait that can be modified through specific training (McCrae and Costa, 1999). These different definitions of mindfulness from diverse perspectives imply that mindfulness is a multidimensional concept. Evaluating mindfulness necessitates adherence to theoretical standards, such as its historical definitions, measurement precision, psychological properties, and hypothesis testing, including assessments of convergent and discriminant validity.

Currently, there is notable diversity in the emphasis of various scales designed to measure mindfulness. This semantic heterogeneity presents challenges within the laboratory setting and may hinder accurate measurement and investigation of mindfulness in the real-world contexts. Furthermore, existing scales do not offer a comprehensive assessment of mindfulness abilities and levels (Brown et al., 2007). For instance, the Mindful Attention Awareness Scale (MAAS) specifically focuses on the attentional aspect of mindfulness. The Kentucky Inventory of Mindfulness Skills (KIMS) and the Five Facet Mindfulness Inventory (FMI) assess mindfulness as a multifaceted concept. However, these facets differ from one another (Baer et al., 2006). Research has indicated that the correlation coefficients of mindfulness among MAAS, Cognitive and Affective Mindfulness Scale-Revised (CAMS), FMI, KIMS, and the Philadelphia Mindfulness Scale (PHLMS) range between 0.21 and 0.67 (Cardaciotto et al., 2008). Variations in the aspects of mindfulness addressed by different tools pose a direct obstacle to the comparability and reproducibility of research findings. In 2006, Ruth Baer and colleagues amalgamated the aforementioned five mindfulness scales. They discerned five distinct and interpretable dimensions through exploratory and confirmatory factor analyses: observation, description, acting with awareness, non-judgment of inner experiences, and non-reactivity to inner experiences. However, these dimensions failed to fully capture all components of mindfulness. Furthermore, the diverse factors and constructs represented by assessment tools mirror different mindfulness skills. At the same time, researchers have considerably enhanced the level of their scrutiny of the favorable effects of mindfulness on mental health (Coffey et al., 2010; Hanley et al., 2015; Omid et al., 2017; Jamieson et al., 2022), physical well-being (Crane et al., 2010a,b), behavioral adaptation (Hapiro et al., 2006; Williams et al., 2011), and wisdom (Wang et al., 2023a,b). Nevertheless, aligning high-dimensional mindfulness skills or abilities with corresponding mindfulness components in existing measurement tools remains challenging. In particular, in the wake of the COVID-19 pandemic, prominently endorsed mindfulness interventions, like Mindfulness-Based Stress Reduction, Mindfulness-Based Cognitive Therapy, mindfulness meditation awareness training, and third-wave therapies with experiential acceptance as the core of the intervention (acceptance commitment therapy, DBT, etc.), have underscored the urgent need of scientifically precise mindfulness assessment tools to gauge the quality and comprehensiveness of these programs.

The Comprehensive Inventory of Mindfulness Experiences (CHIME) is currently the most integrated scale of mindfulness components. It is based on the theoretical framework of mindfulness (Krägeloh et al., 2019; Karl et al., 2024) and integrates all the emphasized elements of mindfulness highlighted by Bergomi et al. (2014). First, the CHIME is composed of 37 items across eight subscales, each designed to meticulously capture the distinct yet interwoven facets of mindfulness, thereby ensuring a holistic appraisal of an individual’s mindfulness capabilities and skills. The Awareness of Internal Experiences subscale gauges attention to one’s internal emotional, cognitive, and sensory landscapes, embodying the introspective facet of mindfulness. The Awareness of External Experiences subscale focuses on an individual’s perception and engagement with the external environment, thereby capturing the extroverted aspect of mindfulness. The Acting with Awareness subscale evaluates the conscious presence in one’s actions, underscoring the importance of living in the present moment and engaging mindfully with tasks. The Acceptance and Non-Judgment subscale measures unconditional acceptance of experiences, thus eschewing the evaluation or labeling, and highlighting the nonjudgmental stance that is intrinsic to mindfulness. The Non-Reactivity and Decentering subscale pertains to the capacity to observe one’s thoughts and emotions without becoming ensnared by them, reflecting the detached observational nature of mindfulness. The Openness to Experiences subscale measures an individual’s willingness to embrace a broad spectrum of experiences, mirroring the open quality of mindfulness. The Relativity of Thoughts and Reality subscale acknowledges the transient nature of thoughts and their non-absolute status as truths, emphasizing the discerning aspect of mindfulness. The Insightful Understanding subscale probes the depths of the insight and comprehension arising from mindfulness practice, which is vital for unlocking its transformative potential. Second, CHIME’s scale design is infused with the concept of mindfulness rooted in Eastern spiritual traditions, particularly highlighting its profound historical and spiritual lineage, with a nod to its foundational role in Buddhism. Within CHIME’s framework, there is a pronounced emphasis on “Awareness of the Relativity of Thoughts and Reality” and “Insightful Understanding,” both of which resonate with the mindfulness precepts of Eastern traditions. “Insight understanding” captures the spiritual quintessence of mindfulness, setting it apart from other scales. According to the developers of this scale, its inclusive domain forges a bridge to the spiritual profundity of mindfulness, accommodating its broader secular utility. Traditional interpretations (Harvey, 2013) regard mindfulness as an instrument for understanding the true nature of reality, alleviating sufferings, and fostering well-being. From this vantage point, the integration of “wisdom factors” becomes indispensable. These elements of wisdom, providing insights into life’s imperfections, impermanence, and non-self, nurture the wisdom and understanding for navigating life’s ephemeral nature. CHIME’s profound understanding and recognition of the relativity of thoughts operationalize these wisdom factors, bridging the gap between traditional measures and concepts, while deepening the understanding of Eastern genesis and Buddhist underpinnings of mindfulness. Lastly, CHIME encompasses a diverse array of mindfulness skills and personal experience characteristics, transcending the limitations of meditation experience, thereby broadening its applicability (Bergomi et al., 2015). An empirical study in Australian adolescents across four samples substantiated an 8-factor, 25-item Adolescent Comprehensive Mindfulness Experience Scale (Johnson et al., 2017). The Dutch rendition of CHIME has successfully undergone validation. Moreover, a concise version, CHIME-SF, has been introduced (Cladder-Micus et al., 2019). CHIME has also demonstrated commendable psychometric properties in New Zealand samples (Medvedev et al., 2018). The English iteration of CHIME has been subjected to rigorous psychometric evaluation with an American sample, affirming its robust measurement properties (Wilkinson et al., 2023).

Mindfulness, as a philosophy of existence, is deeply ingrained in traditional Chinese wisdom, traceable at least to the third patriarch of Chinese Zen, Sengcan, and the sixth patriarch, Huineng, with philosophical echoes in the teachings of Laozi and Zhuangzi. In China, mindfulness practices are widely employed and may exhibit broader reach and distinctive Eastern characteristics compared to other regions. Against this Eastern cultural backdrop, the understanding of mindfulness in China might differ from that in the regions where the scales were originally developed. For instance, the understanding of mindfulness among Chinese individuals is rooted in the Buddhist “Four Foundations of Mindfulness (or Satipatthanas),” which are considered indispensable for cultivating wisdom and attaining enlightenment (Ñanamoli and Bodhi, 1995; Anālayo, 2006). An advanced level of mindfulness is characterized as a form of spiritual awakening and enlightenment, representing the pursuit of psychological well-being that is self-actualizing and harmoniously connected with others, rather than the pursuit of subjective, fleeting hedonistic happiness. Given the aforementioned advantages of CHIME in adopting Eastern spiritual traditional concepts of mindfulness and its ability to comprehensively capture the eight crucial aspects of mindfulness experiences, this study aims to translate and adapt CHIME into a version more attuned to the Chinese cultural context. The study examines the psychometric properties of this scale among Chinese university students and evaluates its reliability and validity among the Chinese populace, with the goal of establishing a scientifically robust measurement tool for mindfulness research and practice in China.

## 2 Methods

### 2.1 Participants

We initially recruited 410 university students from a university in Hubei Province, China, for a pilot survey to assess potential issues with the wording of the questionnaire and finalize its content. The survey was conducted through on-site paper-and-pencil testing, administered by psychology graduate students who had undergone specialized training, and was carried out in a class setting. Overall, 372 valid scales were collected.

Sample 1: For the formal assessment, the participants were split into two groups. Using a convenience sampling method, the first group underwent on-site paper-and-pencil testing. Following the same testing procedure as in the pilot survey, students from four universities in Jiangsu, Gansu, Sichuan, and Hubei were chosen as participants. The second group engaged in online testing, recruiting university students to complete a questionnaire with identical content to the paper version. A total of 2,113 scales were distributed and collected. We meticulously excluded invalid responses, which included patterned answers with repetitive or regular response patterns to ≥10 questions, responses with a completion time of 400 s or less, responses that were inconsistent, incomplete surveys, and technical errors that occurred during the survey administration. Finally, we obtained 1,785 valid scales. Consequently, the effective completion rate of the entire set of qualified scales reached 84.4%.

Sample 2: Two weeks following the formal assessment, a subset of participants was chosen for retesting the utilized paper-and-pencil method. Accordingly, 490 scales were distributed on-site. After eliminating unmatched data, 391 valid scales were obtained.

### 2.2 Measures

#### 2.2.1 Comprehensive inventory of mindfulness experiences

The CHIME-37 questionnaire comprised 37 items, encompassing eight subscales (Bergomi et al., 2013): (1) Awareness of Internal Experiences, (2) Awareness of External Experiences, (3) Acting with Awareness, (4) Acceptance and Non-Judgment, (5) Decentering and Non-reactivity, (6) Experiential Openness, (7) Relativity of Thoughts and Reality, and (8) Insightful Understanding. Each item was rated on a Likert scale ranging from 1 (never) to 7 (always). All samples utilized the CHIME questionnaire.

We adhered to the guidelines of the stage model for the cross-cultural adaptation of assessment tools proposed by Geisinger (1994) to translate the original version of CHIME into Chinese.

In the initial stage, a bilingual individual proficient in German and Chinese translated the CHIME items into Chinese. This individual, with 10 years of experience in mindfulness practice, possessed a profound understanding of the concepts. In the second stage, two bilingual individuals, fluent in German and Chinese and experienced in mindfulness practice, collaboratively assessed the initial translation. The evaluation aimed to ensure consistency with the original text and the comprehensibility of the translated version. After a joint review of the translation, the evaluators provided feedback to the translator in the third stage. Following this feedback, the translator revised the draft of the Chinese CHIME based on the evaluators’ suggestions. Any inconsistencies throughout the process were discussed and modified for proper alignment, ensuring that the expressions maintained the original German meaning while being clear and understandable.

In the fourth stage, the authors introduced the initial draft of the Chinese CHIME to a small sample (*n* = 372). Their characteristics were similar to those of the final study sample (e.g., university undergraduates). Consistent with Geisinger’s suggestions, the participants from the convenience sample were interviewed by researchers to understand their experiences regarding the comprehensibility, wording, and understanding of the items. Drawing from participants’ feedback and response patterns, relevant issues regarding the questionnaire content were identified. The research team engaged in discussions to address these issues, leading to minor adjustments in the translation draft. Subsequent to these modifications, the final version of the Chinese CHIME was established.

#### 2.2.2 Satisfaction with life scale

The Satisfaction with Life Scale (SWLS), developed by Diener et al. (1985), was used to assess life satisfaction. The Chinese version of the SWLS has been utilized in previous large-scale cross-sectional studies (Song et al., 2013; Kong et al., 2014). The confirmatory factor analysis (CFA) indicated that this scale demonstrated a good fit: *χ*^2^/df = 2.859, RMSEA = 0.044, CFI = 0.997, TLI = 0.953, and SRMR = 0.010. The participants responded on a 7-point Likert scale (1 = strongly disagree, 7 = strongly agree), with higher scores indicating a greater subjective sense of well-being. The Cronbach’s *α* coefficient for the study sample was 0.838.

#### 2.2.3 Psychological well-being

The Flourishing Scale (FS), comprising eight items, was used to assess psychological well-being (PWB) (Diener et al., 2010). The Chinese version of the FS has been validated in the community and adolescent samples (Duan and Xie, 2019). The CFA indicated that this scale demonstrated an acceptable fit: *χ*^2^/df = 3.322, RMSEA = 0.054, CFI = 0.995, TLI = 0.993, and SRMR = 0.008. The participants responded on a 7-point Likert scale (1 = strongly disagree, 7 = strongly agree), with higher scores indicating a greater sense of PWB. The Cronbach’s *α* coefficient was 0.896.

#### 2.2.4 Peace of mind

This scale comprised seven items describing the respondents’ sense of internal peace and ease in their daily life (Lee et al., 2013). The CFA demonstrated a good fit of this scale: *χ*^2^/df = 3.770, RMSEA = 0.060, CFI = 0.962, TLI = 0.953, and SRMR = 0.030. Sample items included the following: 1. My mind is free and at ease, and 4. I have peace and harmony in mind. The participants were asked to indicate the frequency of their feelings on a scale from 1 (Not at all) to 5 (All of the time). Peace of mind was assessed based on the sum of scores of the seven items, with a high score indicating a high level of peace of mind. The Cronbach’s alpha coefficient was 0.916.

#### 2.2.5 SicknessQ

SicknessQ, a concise tool comprising nine items, was utilized to evaluate the perceived sickness behavior of individuals (Andreasson et al., 2018; Tang et al., 2022). The CFA demonstrated the scale’s good fit: *χ*^2^/df = 4.907, RMSEA = 0.064, CFI = 0.976, TLI = 0.965, and SRMR = 0.030. The participants were required to assess their current feelings using a four-level scale ranging from 0 to 3 (0 = disagree, 1 = somewhat agree, 2 = mostly agree, 3 = agree), with higher scores indicating a lower overall level of physical and mental health for individuals. In this study, the overall questionnaire and each dimension exhibited Cronbach’s alpha coefficients of 0.847, 0.768, and 0.848, respectively.

#### 2.2.6 Depression, anxiety, and stress scale

This scale was used to assess the participants’ psychological health. It has proven to be a reliable and effective measure for evaluating mental well-being of the Chinese population (Wang et al., 2016). The CFA indicated an acceptable fit of the scale: *χ*^2^/df = 3.513, RMSEA = 0.052, CFI = 0.957, TLI = 0.951, and SRMR = 0.027. Item scores were recorded using a 4-point Likert scale (1 = strongly disagree, 5 = strongly agree), with higher scores indicating higher levels of depression, anxiety, and stress for individuals. The overall questionnaire and each dimension demonstrated Cronbach’s alpha coefficients of 0.898, 0.917, 0.895, and 0.889, respectively.

#### 2.2.7 Self-reflection and insight scale

This scale, developed by Grant et al. (2002) and Liu et al. (2018), comprised 20 self-assessment items and three subscales of Engagement Reflection, Motivation Reflection, and Insight. The CFA indicated an acceptable fit of the scale: *χ*^2^/df = 1.189, RMSEA = 0.014, CFI = 0.997, TLI = 0.997, and SRMR = 0.016. The items were scored using a Likert 6-point scale (1 = Strongly Disagree, 6 = Strongly Agree). Higher scores reflect higher levels of reflection and insight in an individual. In this study, the overall questionnaire and each dimension had Cronbach’s *α* coefficients of 0.944, 0.954, and 0.893, respectively.

#### 2.2.8 Emotion regulation questionnaire

This questionnaire, developed by Gross (1998) and Wang et al. (2007), comprised 10 items assessing two dimensions: Cognitive Reappraisal and Expressive Suppression. The CFA indicated an acceptable fit of the scale: *χ*^2^/df = 1.254, RMSEA = 0.060, CFI = 0.999, TLI = 0.998, and SRMR = 0.016. Item scores were recorded on a 7-point Likert scale (1 = strongly disagree, 7 = strongly agree), with higher scores indicating a higher frequency of employing emotion regulation strategies. The Chinese version of the questionnaire demonstrated robust reliability and validity. The Cronbach’s alpha coefficients for each dimension of the questionnaire were 0.917 and 0.923, respectively.

### 2.3 Data analysis

Utilizing SPSS 22.0, we conducted the item, exploratory factor, internal consistency reliability, criterion-related validity, and test–retest reliability analyses. CFA was performed using MPLUS 8. Values below 0.05 were considered statistically significant. Furthermore, exact *p* values were reported to signify the level of significance in the findings.

### 2.4 Ethical considerations

Ethical considerations were paramount, ensuring participants’ well-being and rights. The study was ethically approved by Central China Normal University’s committee, emphasizing adherence to guidelines and transparency. The participants provided informed consent, understanding the study’s purpose, procedures, risks, and benefits, with the option to withdraw freely. Privacy and confidentiality were strictly maintained, with data anonymization to protect anonymity. Emotional well-being was safeguarded through debriefing and support resources. The study prioritized participants’ welfare, following ethical guidelines to protect their rights and ensure research integrity.

## 3 Results

In the pilot survey sample, 410 subjects completed the CHIME-37. Among them, there were 162 male participants (43.5%) and 210 female participants (56.5%). The average age of the participants was 19.21 ± 0.63 years. For the formal assessment, 819 (45.9%) male participants and 966 (54.1%) female participants were included, with an average age of 20.17 ± 1.62 years. These participants were split into two groups, and Table 1 presents their demographic characteristics. In the overall test–retest, 140 were male participants and 231 were female participants, accounting for 40.9 and 59.1%, respectively. The average age of these participants was 19.53 ± 0.76 years.

**Table 1:** Descriptive statistics of the participants.

Group		*n*	%	Age (Mean ± SD)
1	1. Gender			
	Male	398	47.49%	19.98 ± 1.59
	Female	440	52.51%	20.07 ± 1.60
	2. Mindfulness practice			
	Yes	307	36.63%	19.99 ± 1.62
	No	531	63.37%	20.05 ± 1.58
2	1. Gender			
	Male	428	45.2	19.88 ± 1.50
	Female	519	54.8	19.99 ± 1.49
	2. Mindfulness practice			
	Yes	360	38.0	19.86 ± 1.35
	No	587	62.0	19.90 ± 1.57

### 3.1 Item analysis

The critical ratio method was utilized for the item analysis of the questionnaire. The CHIME-37 item scores were arranged in descending order, with the bottom 27% of participants categorized as the low-scoring group and the top 27% as the high-scoring group. We examined disparities between the two groups on each item. Independent samples t-tests were conducted for each item’s scores, revealing significant differences for all items (*p* < 0.001). Furthermore, correlation analyses were performed between the scores of each item and the total score. The results indicated correlation coefficients ranging between 0.417 and 0.734, all statistically significant at 0.01 level. Further details are presented in Table 2.

**Table 2:** The overall correlation and decision values for each item of the CHIME-37.

Items	*t*	*r*	Items	*t*	*r*
1	18.005**	0.603**	20	16.577**	0.578**
2	15.710**	0.513**	21	12.451**	0.432**
3	15.896**	0.540**	22	13.550**	0.504**
4	13.869**	0.501**	23	16.971**	0.551**
5	17.434**	0.575**	24	19.669**	0.598**
6	16.681**	0.561**	25	16.022**	0.556**
7	15.494**	0.552**	26	15.953**	0.539**
8	20.648**	0.629**	27	18.981**	0.584**
9	16.051**	0.554**	28	18.221**	0.612**
10	16.141**	0.534**	29	11.053**	0.385**
12	18.309**	0.590**	30	14.269**	0.538**
12	21.340**	0.650**	31	16.448**	0.561**
13	16.842**	0.591**	32	12.350**	0.422**
14	13.734**	0.514**	33	19.038**	0.608**
15	19.387**	0.604**	34	13.345**	0.525**
16	19.159**	0.612**	35	15.450**	0.542**
17	15.600**	0.556**	36	15.524**	0.541**
18	11.382**	0.393**	37	18.335**	0.595**
19	21.284**	0.621**			

**p < 0.01.

### 3.2 Structural validity

#### 3.2.1 Exploratory factor analysis

**Figure 1:**
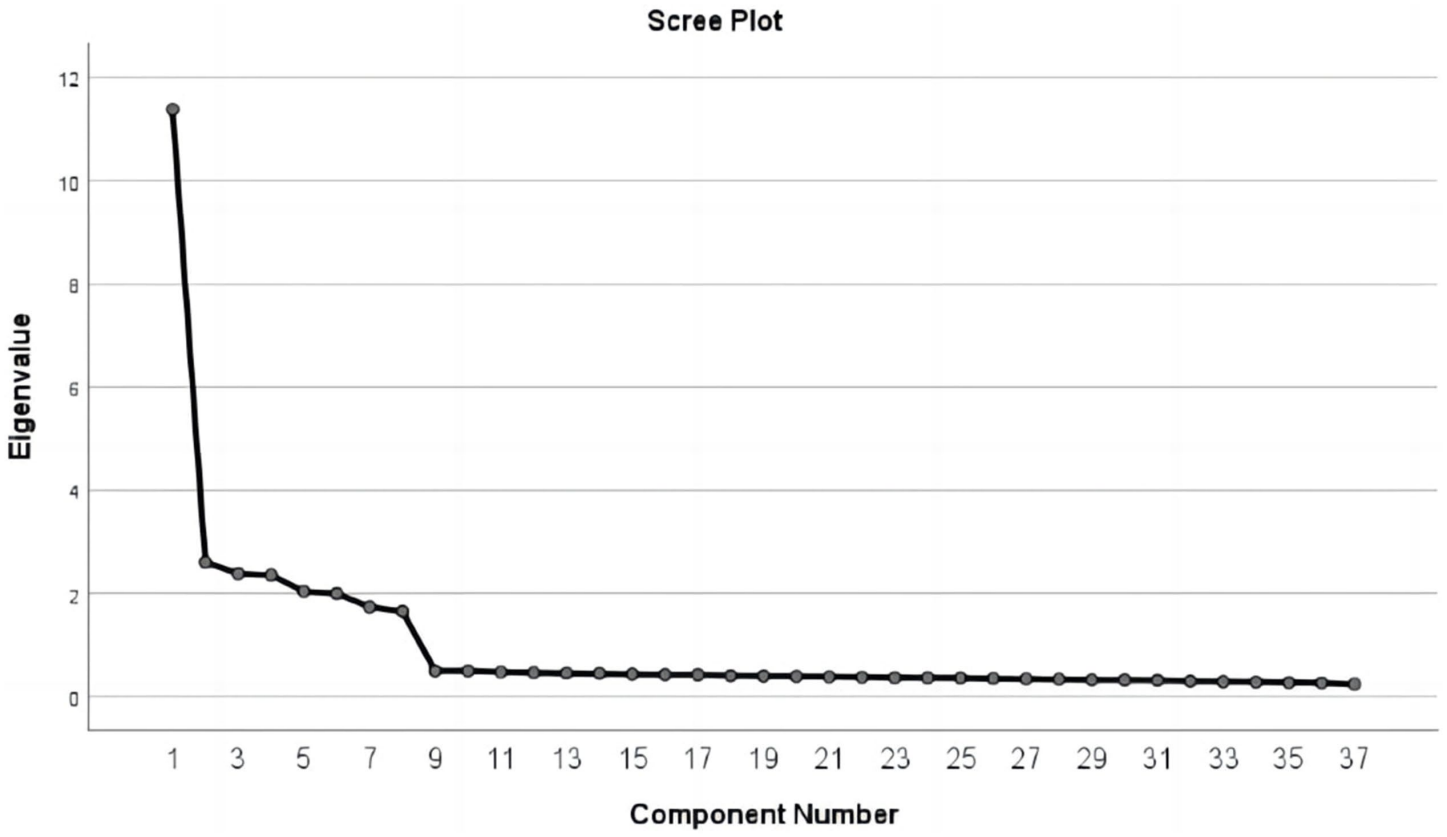
Scree plot.

The Chinese version of CHIME-37 underwent exploratory factor analysis (EFA) using data from Group 1 (*n* = 838). EFA was employed with the principal component analysis and varimax rotation to assess conformity levels and assign names to the extracted factors in the internal structure of CHIME among Chinese college students. The Kaiser–Meyer–Olkin value was 0.940, and the Bartlett sphericity test yielded *χ*^2^ = 17,700.459 (df = 666, *p* < 0.001), indicating the suitability of the data for EFA. Factor extraction retained factors with eigenvalues greater than 1, resulting in a cumulative variance contribution of 70.696%. This reflects substantial explanatory power of the factors, preserving the original data information comprehensively. The variance percentage of the first factor was 30.754, which is less than 40%, suggesting the absence of severe common method bias. Moreover, the analysis of the scree plot (Figure 1) led to the decision to extract eight factors. Although the 7-factor and 8-factor models demonstrated good-to-excellent fits, model fit indices indicated a superior fit for the 8-factor model. The chi-square difference test was significant, supporting the adoption of the more parsimonious (8-factor) model, as outlined in Table 3. Factor loadings of the items illustrated a better conceptual fit with the 8-factor model. The factor loadings and communality for the 37 items are presented in Table 4. In line with the nomenclature proposed by Bergomi et al., these eight factors were named as follows: Awareness of Internal Experiences, Awareness of External Experiences, Acting with Awareness, Acceptance and Non-Judgment, Decentering and Non-Response, Openness to Experiences, Relativity of Thoughts, and Insightful Understanding.

**Table 3:** Exploratory factor analyses: model fit comparisons.

Model	RMSEA	CFI	TLI	*χ* ^2^	df	Models compared	*χ* ^2^	df
1-factor	0.114	0.673	0.653	8,319.78	629			
2-factor	0.105	0.723	0.707	7,126.564	628	1-factor against 2-factor	1,193.216	1
3-factor	0.098	0.758	0.743	5,305.354	626	2-factor against 3-factor	1,821.21	2
4-factor	0.086	0.813	0.801	5,004.739	623	3-factor against 4-factor	300.615	3
5-factor	0.078	0.846	0.835	4,230.981	619	4-factor against 5-factor	773.758	4
6-factor	0.072	0.871	0.86	3,648.039	614	5-factor against 6-factor	582.942	5
7-factor	0.052	0.934	0.927	2,164.599	608	6-factor against 7-factor	1,483.44	6
8-factor	0.028	0.981	0.979	1,052.624	601	7-factor against 8-factor	1,111.975	7

RMSEA, root-mean-square error of approximation; CFI, comparative fit index; TLI, Tucker–Lewis index. All *p* < 0.001.

**Table 4:** Exploratory factor analysis of CHIME-37 with item-factor loadings and proportion of communality (*N* = 838).

Items	Factor loadings								Communality
	F1	F2	F3	F4	F5	F6	F7	F8	
F1: Awareness of internal experiences									
1	0.740								0.671
5	0.771								0.689
14	0.756								0.679
29	0.759								0.699
34	0.765								0.703
F2: Awareness of external experiences									
9		0.789							0.724
18		0.795							0.737
21		0.779							0.730
27		0.803							0.740
F3: Acting with awareness									
10			0.810						0.714
12			0.778						0.713
17			0.769						0.714
26			0.803						0.726
38			0.758						0.690
F4: Acceptance and non-judgment									
2				0.782					0.693
7				0.768					0.700
32				0.785					0.730
36				0.780					0.712
F5: Decentering and non-response									
8					0.784				0.717
13					0.759				0.707
16					0.807				0.731
20					0.738				0.661
25					0.764				0.681
28					0.790				0.698
F6: Openness to experiences									
19						0.826			0.714
22						0.834			0.742
30						0.827			0.714
33						0.834			0.739
F7: Relativity of thoughts and reality									
4							0.782		0.688
23							0.794		0.710
31							0.769		0.697
35							0.774		0.692
F8: Insightful understanding									
3								0.762	0.664
6								0.795	0.719
15								0.816	0.719
24								0.787	0.706
37								0.786	0.695

#### 3.2.2 Confirmatory factor analysis

**Figure 2:**
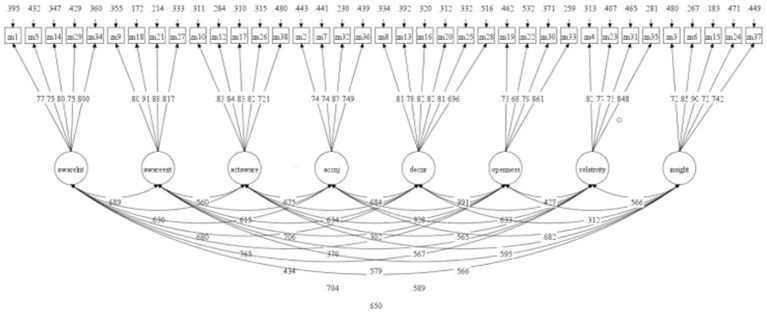
Standardized parameters of the 8-factor model of the CHIME.

CFA was performed on the structure of the Chinese version of CHIME-37 by using Sample 2 (*n* = 947). Initially, we established an 8-factor model based on the factor structure of the original questionnaire and conducted structural validation for the Chinese version to assess its applicability. The original factor structure included the following: (1) Awareness of Internal Experiences (items 1, 5, 14, 29, and 34); (2) Awareness of External Experiences (items 9, 18, 21, and 27); (3) Acting with Awareness (items 10, 12, 17, 26, and 38); (4) Acceptance and nonjudgmental attitude (items 2, 7, 32, and 36); (5) Non-Reactivity and Decentering (items 8, 13, 16, 20, 25, and 28); (6) Openness to Experiences (items 19, 22, 30, and 33); (7) Relativity of Thoughts and Reality (items 4, 23, 31, and 35); and (8) Insightful Understanding (items 3, 6, 15, 24, and 37). The results of the CFA revealed that the fit indices for the 8-factor model were *χ*^2^/df = 1.751, CFI = 0.981, TLI = 0.979, SRMR = 0.027, and RMSEA = 0.028, indicating a relatively good fit. This finding suggests that the original factor structure is also applicable to the Chinese version of the questionnaire. Figure 2 depicts standardized parameters of the 8-factor model of the CHIME-37.

#### 3.2.3 Internal validity analysis

A CFA of the CHIME questionnaire was conducted. Internal validity indicators, inter-dimension correlations, and convergent validity were computed. Table 5 presents the results in detail. The analysis revealed significant correlations between CHIME and its dimensions, with Pearson correlation coefficients ranging between 0.302 and 0.704. All pairwise correlations between CHIME dimensions were statistically significant, with the average variance extracted (AVE) value exceeding 0.5 for each dimension. This finding suggests that the CHIME questionnaire could effectively capture various facets of mindfulness, demonstrating robust internal discriminant validity.

**Table 5:** Correlation coefficients and average variance extracted values among dimensions of the Chinese version of CHIME-37.

Dimensions	1	2	3	4	5	6	7	8
1: Awarelnt	0.780							
2: AwareExt	0.689**	0.856						
3: ActAware	0.630**	0.560**	0.812					
4: AccNJ	0.680**	0.615**	0.675**	0.782				
5: DecNR	0.765**	0.706**	0.634**	0.684**	0.796			
6: Openness	0.434**	0.370**	0.302**	0.328**	0.391**	0.771		
7: Relativity	0.704**	0.579**	0.567**	0.565**	0.633**	0.427**	0.796	
8: Insight	0.650**	0.589**	0.566**	0.595**	0.682**	0.312**	0.566**	0.794

**p < 0.01. The average variance extracted values are provided on the diagonal.

#### 3.2.4 Criterion-related validity analysis

We conducted Pearson’s correlation analyses to examine the relations between the total score and each dimension of CHIME and scores on various factors. These factors included subjective well-being, psychological well-being, mental tranquility, physical and mental health, depression-anxiety-stress, self-reflection and insight, cognitive reappraisal, and expressive suppression. The total mindfulness experience score and its dimensions exhibited significant positive correlations with subjective well-being, psychological well-being, mental tranquility, and cognitive reappraisal (*p* < 0.01). Furthermore, the total mindfulness experience score and its dimensions exhibited negative correlations with physical and mental health, depression-anxiety-stress, and expressive suppression (*p* < 0.01) (Refer to Table 6 for details).

**Table 6:** Analysis of the calibration-related validity of CHIME-37.

	Awarelnt	AwareExt	ActAware	AccNJ	DecNR	Openness	Relativity	Insight	CHIME
Subjective well-being	0.288**	0.291**	0.280**	0.296**	0.330**	0.246**	0.256**	0.322**	0.380**
Psychological well-being	0.419**	0.412**	0.400**	0.407**	0.429**	0.291**	0.338**	0.384**	0.508**
Peace of mind	0.439**	0.377**	0.381**	0.365**	0.412**	0.167**	0.368**	0.325**	0.470**
Physical condition	−0.230**	−0.176**	−0.237**	−0.299**	−0.215**	−0.238**	−0.221**	−0.190**	−0.293**
Psychological status	−0.214**	−0.145**	−0.257**	−0.267**	−0.208**	−0.193**	−0.166**	−0.159**	−0.264**
Psychosomatic illness	−0.242**	−0.173**	−0.271**	−0.307**	−0.231**	−0.233**	−0.208**	−0.189**	−0.303**
Stress	−0.437**	−0.377**	−0.454**	−0.420**	−0.443**	−0.296**	−0.385**	−0.373**	−0.526**
Anxiety	−0.376**	−0.373**	−0.416**	−0.390**	−0.388**	−0.265**	−0.313**	−0.338**	−0.472**
Depression	−0.341**	−0.331**	−0.418**	−0.400**	−0.377**	−0.251**	−0.329**	−0.362**	−0.463**
Depression-anxiety-stress	−0.532**	−0.496**	−0.592**	−0.556**	−0.557**	−0.374**	−0.474**	−0.494**	−0.673**
Self-reflection	0.377**	0.341**	0.364**	0.377**	0.413**	0.244**	0.316**	0.315**	0.455**
Insight	0.323**	0.283**	0.351**	0.348**	0.337**	0.253**	0.276**	0.310**	0.409**
Self-reflection and insight	0.407**	0.365**	0.409**	0.418**	0.440**	0.281**	0.344**	0.356**	0.499**
Cognitive reappraisal	0.402**	0.365**	0.337**	0.339**	0.383**	0.284**	0.312**	0.295**	0.448**
Expression inhibition	−0.493**	−0.426**	−0.498**	−0.436**	−0.505**	−0.354**	−0.417**	−0.463**	−0.594**

**p < 0.01.

#### 3.2.5 Reliability analysis

The overall Cronbach’s *α* coefficient for the questionnaire was 0.961, with individual dimensions ranging between 0.883 and 0.961. The overall test–retest reliability coefficient of the questionnaire was 0.840 (*p* < 0.01), and test–retest reliability coefficients for individual dimensions ranged between 0.746** and 0.732** (Ps < 0.01). When dividing the questionnaire into two equal halves based on item numbers and correlating the scores of the two halves, the split-half reliability for the overall questionnaire was 0.871, with individual dimensions ranging between 0.795 and 0.919 (Refer to Table 7 for the results of the reliability analysis).

**Table 7:** Descriptive statistics, test–retest reliability, split-half reliability, and internal consistency reliability results.

Dimension	M ± SD	Retest reliability	Split-half reliability	*α*
Awarelnt	24.48 ± 5.23	0.746**	0.853**	0.883
AwareExt	20.43 ± 4.69	0.848**	0.919**	0.914
ActAware	20.10 ± 5.59	0.885**	0.856**	0.905
AccNJ	16.71 ± 4.34	0.840**	0.802**	0.855
DecNR	26.13 ± 6.30	0.825**	0.898**	0.910
Openness	17.46 ± 4.13	0.861**	0.891**	0.848
Relativity	18.86 ± 3.98	0.856**	0.871**	0.871
Insight	23.11 ± 5.14	0.886**	0.795**	0.891
CHIME	167.28 ± 30.20	0.840**	0.894**	0.961

### 3.3 Measurement invariance test

Following the recommendation of Cheung and Rensvold (2002), we employed ΔCFI ≤ 0.01 and ΔRMSEA ≤ 0.015 as criteria for assessing invariance. The results, presented in Table 8, indicate that the metric and scalar invariance hypotheses hold true within the gender subgroup. In the subgroup based on the extent of mindfulness practice, the metric and scalar invariance hypotheses were validated. These findings suggest that the metric and scalar invariance hypotheses are supported within the identified subgroups.

**Table 8:** Measurement invariance test.

Model	Chi-square	df	CFI	RMSEA (90% CI)	ΔCFI	ΔRMSEA
Gender						
Configural	1,638.994	1,202	0.981	0.028 (0.024, 0.031)		
Metric	1,658.591	1,231	0.982	0.027 (0.024, 0.030)	0.001	0.001
Scalar	1,684.576	1,260	0.982	0.027 (0.023, 0.030)	0.000	0.000
Mindfulness practice						
Configural	1,682.938	1,202	0.980	0.029 (0.026, 0.032)		
Metric	1,710.768	1,231	0.980	0.029 (0.025, 0.032)	0.000	0.000
Scalar	1,748.715	0260	0.979	0.029 (0.025, 0.032)	0.001	0.000

## 4 Discussion

This study, for the first time, investigated the suitability of a multifactorial mindfulness tool for Chinese university students. Following standard procedures, the CHIME questionnaire was translated into Chinese language and administered to Chinese university students to assess CHIME-37. The results showed the reliability of the 8-factor, 37-item mindfulness measurement tool (CHIME-37) among Chinese university students. In addition, the scale demonstrated excellent model fit indices and good internal consistency for the total score and eight subscales, consistent with the theoretical structure of the original scale.

Differentiation among the eight dimensions is a crucial characteristic of the CHIME. First, the AVE values for each of the eight dimensions exceeded 0.5, indicating good discriminant validity among the internal dimensions of the Chinese version of CHIME. This finding suggests that the eight skills and abilities of mindfulness are clearly distinguishable from each other. Second, the Pearson’s correlation coefficients among the eight dimensions ranged between 0.3 and 0.7, indicating that these dimensions are correlated but not overlapping. This finding aligns with the theoretical construction of mindfulness, which posits that mindfulness encompasses eight distinct components or skills. This feature holds significant value for the comprehensive and accurate measurement of mindfulness levels as well as the assessment of the completeness and quality of mindfulness programs, which could provide evidence for the effectiveness of mindfulness interventions (Haenen et al., 2016). Furthermore, mindfulness demonstrated moderate correlations with the criterion-related validity. Thus, CHIME has a strong predictive power in measuring subjective well-being, psychological well-being, emotion regulation, insight, anxiety, depression, perceived stress, and overall health. In the analysis of criterion-related validity, subscales of the CHIME encompassing its eight dimensions exhibited significant positive correlations with subjective well-being, psychological well-being, emotional tranquility, self-reflection, and insight, while they showed negative correlations with physical and mental health issues, depression-anxiety-stress, and expressive suppression. These findings suggest that the mindfulness skills and abilities are centered on redirecting individuals’ attention to directly experiencing their bodies, minds, emotions, sensations, and thoughts with unconditional acceptance. This process can help college students avoid comparison from peers in terms of achievements or other aspects, thereby fostering inner peace, happiness, enhanced reflection and insights, and improved cognitive regulatory abilities. Concurrently, mindfulness skills and abilities can alleviate physical and mental sufferings, reduce perceived stress levels, and minimize emotional suppression. The results indicate that a decline in mindfulness may be associated with the deterioration in physical health, psychological distress, and emotional imbalance, consistent with previous research findings (Baer et al., 2012).

The Chinese version of the CHIME demonstrated satisfactory test–retest reliability, split-half reliability, and internal consistency. Contrary to Johnson et al.’s revised version of the CHIME, which displayed good internal reliability for individual subscales, but not for the total score, and thus not recommended for use (Johnson et al., 2017), the Chinese version of the CHIME demonstrated good internal reliability for both the individual subscales and the total score. Consequently, both the overall scale and its subscales are deemed usable.

Previous cross-cultural research has indicated that hedonic well-being, as measured by SWB, only partially reflects the true state of well-being among Eastern populations (Ho et al., 2014). By contrast, eudaimonic well-being, measured by PWB, encompasses various relationships emphasized in traditional Eastern cultures (kinship and friendships), social sharing, and self-actualization, making it suitable for assessing the welfare of Chinese individuals. In this study, mindfulness exhibited a significantly stronger correlation with PWB than with subjective well-being (SWLS, hedonic well-being), confirming that mindfulness is more closely linked to the aspects of well-being related to self-actualization, such as autonomy, personal growth, and life goals (Bauer et al., 2005), as well as self-acceptance, mastery of one’s environment, and positive relationships with others. This result is consistent with the collectivist cultural context of China. In Eastern cultures, mindfulness focuses on the realization of human potential and harmonious relationships. In future mindfulness intervention studies using CHIME, PWB might be a more suitable predictive indicator for Eastern populations because it encompasses closer relationships, greater self-acceptance, and deeper spirituality, leading to increased SWB and personal growth (cf. Ryff and Singer, 1996; van Dierendonck, 2004). Individuals’ college years or early adulthood is a critical period for the development of wisdom (Jordan, 2005), and mindfulness may contribute to enhancing wisdom and insights. In this study, these skills or abilities considered as crucial mindfulness traits in Eastern culture (factors 7 and 8) correlated strongly with reflection and insights, excellently validating this point. Therefore, the revised CHIME utilized in this study is appropriate for the cultural context in China.

We revised the scale’s items in this study in line with Silva’s emphasis on the importance of conducting in-depth research on the application of mindfulness across various cultures and disciplines (Silva, 2015). For example, discrepancies were identified between the items of the revised Chinese version of Factor 3 (Acting with Awareness) and Factor 4 (Acceptance and Non-Judgment) with their original German version. The original item corresponding to Factor 3, “When reading, I have to read certain passages repeatedly because I am thinking about other things,” was reframed to prevent misunderstanding; this construction has the potential for misinterpretation as the initial half of the question was based on the Chinese cultural principle that “the meaning of a book is revealed after reading it a hundred times,” whereas the latter half shared similarities with the right Chinese approach to reading that emphasizes “imagination and drawing inferences from one another.” The revised item is as follows: “While engaged in activities, my focus frequently wanders, and I find myself readily susceptible to distractions.” Similarly, the original statements for two items of Factor 4 (Acceptance and Non-Judgment) were “When I make mistakes, I will be strict with myself” and “I face up to my mistakes and difficulties without self-blame.” These statements were likely to be misinterpreted with the introspective and self-effacing nature of Chinese culture, which often involves “introspection, self-blame, and self-punishment.” Hence, in the revised questionnaire, the former item was eliminated, whereas the latter statement was rephrased to “When facing failures and difficulties in life, work, or study, I can forgive my mistakes, gently tolerate myself, and avoid self-denial,” thereby reflecting the aspect of self-acceptance that is valued in Chinese culture and minimizing the risk of comprehension bias during the subjects’ engagement with the survey.

In the CHIME theoretical framework, mindfulness is considered to comprise eight components, representing eight skills or abilities, which is partially consistent with the previous human-centered analysis method that divides mindfulness into three broad categories, ranging from low to high, namely awareness (including the basic dimensions of mindfulness), the regulation of behavior (including aware actions and different responses to inner experiences), and the assessment of wisdom (the most advanced mindfulness skills) (Lilja et al., 2013). In this study, Factor 1 (Awareness of Internal Experiences) and Factor 2 (Awareness of External Experiences) refer to the basic skills of mindfulness, mainly training individuals may be more focused on the feelings and actions of the moment, from “auto navigation mode” to “being mode” (Kabat-Zinn, 1994), and better adapt to adversity and challenges. Factor 3 (Acting with Awareness), Factor 4 (Acceptance and Non-Judgment), Factor 5 (Decentering and Non-Response), and Factor 6 (Openness to Experiences) belong to the middle-order skills of mindfulness behavior regulation. In this study, the middle-order skills of mindfulness include cognitive and emotional regulation, which indirectly affect behavioral regulation. Behavioral regulation is reflected mainly in Factor 3 (Acting with Awareness), that is, maintaining mindfulness in daily life, as well as having focused action and higher executive power. Cognitive adjustment is reflected in Factor 4 (Acceptance and Non-Judgment), that is, when facing failure and frustration, individuals should focus on self-compassion, give themselves a space to accept and contain themselves, and maintain rational mind, emotions, and behaviors. Emotional regulation is reflected in Factor 5 (Decentering and Non-Response) and Factor 6 (Openness to Experiences), that is, in the face of painful emotions, feelings, experiences, and ideas, individuals can let go of attachment, do not struggle, surrender, or resist, remain open to any experience awareness or meta-consciousness, and do not avoid pain. Factor 7 (Relativity of thought and reality) and Factor 8 (Insightful thinking), that is, cognitive defusion, are the higher-order skills or ability of a mindful, intelligent mind; the cognitive integration to get rid of negative thoughts, emotions, and needs in a metacognitive mode; and realize the imperfection, impermanence, and selflessness of life, as well as the wisdom and insights to face the impermanence of life with self-transcendence.

Combined with the results, this study determined that the eight components of CHIME can predict beneficial psychological, functional, physical, and stress-related outcomes among individuals, suggesting that the eight components constitute a potential mechanism through which mindfulness affects individuals. These eight components are in line with Hölzel’s four effective components of mindfulness, namely (a) Regulation of Attention, (b) Awareness of body, (c) Emotional regulation (including reappraisal and exposure, extinction, and reconsolidation), and (d) Change in perspective on the self (Hölzel et al., 2011). The overlapping aspects encompass “Regulation of Attention” and CHIME’s “Conscious Action,” with both emphasizing the aspect of attentiveness; “Awareness of the body” correlates with CHIME’s “Awareness of Internal Experiences” and “Awareness of External Experiences,” highlighting the experiential awareness of the body; Emotional regulation closely aligns with CHIME’s “Acceptance and Non-Judgment” and “Openness to Experiences,” both emphasizing an accepting and open attitude toward experiences. The aspect of “Change in perspective on the self” is closely related to CHIME’s “Awareness of the Relativity of Thoughts and Reality” and “Insightful Understanding,” with all three emphasizing the transformation in one’s self-perception.

## 5 Implications for research and practice

The eight factors of mindfulness correspond to eight skills or abilities associated with mindfulness, positively contributing to enhancing college students’ psychological resilience, mental qualities, emotional regulation, and wisdom. This has implications for improving mental health among college students. Generally, these eight skills and abilities of mindfulness are acquired progressively, beginning with the basic skills and gradually advancing to intermediate and advanced skills. Factors 1 and 2 signify the fundamental dimensions of mindfulness (internal sensations), encompassing basic mindfulness skills. Individuals can nurture the ability to stay connected with their inner and outer experiences through body scanning and breath observation. Factors 3 and 4 can be enhanced through mindful exercises such as walking, stretching, sitting meditation (non-selective awareness practices), and mindful dishwashing. Factors 5 and 6 foster psychological resilience, emotional acceptance, and adaptability to the present moment experiences. These skills can be acquired through meditation on “recognizing aversion,” and the three-minute breath space exercise can contribute to these skills. Factors 7 and 8 help develop individuals’ ability to maintain clear cognition, benevolence, and take wise and insightful actions in response to life’s challenges. These skills can be developed through mindfulness meditation (recognizing the relativity of thoughts and reality) and compassionate meditation, which can contribute to developing these advanced skills. College students engaging in mindfulness practices for the first time can start with basic exercises and progressively move on to intermediate and advanced practices. After establishing a solid foundation in basic skills, they can gradually explore more advanced practices, following a step-by-step approach starting from easy tasks and then switching to challenging tasks.

The Buddhist mental model of mindfulness emphasizes that the accumulation of even a little insight can lead to lasting and highly beneficial effects (Grabovac et al., 2011a). In CHIME, the measurement of the Eastern spiritual tradition is very interesting. For example, “I realize that my mind can change” and “I burst into laughter when I realized that my subjective interpretation would make a big deal out of a simple problem.” These statements are simple and easy to understand, and yet they can capture subtle changes in the heart of participants, which may have clinical value in practice. In Theravada Buddhism, this insight skill can be gradually developed through meditation (Grabovac et al., 2011b), which could be a useful complement to the clinical value of mindfulness interventions, allowing appropriately trained clinicians to guide patients through these practices.

## 6 Limitations and future research

First, the participants were recruited specifically from four universities in the southern, central, and western regions of China, potentially limiting the generalizability of the results to other universities across the country. Future research could address this limitation by seeking a more representative sample from various regions nationwide, particularly from Eastern and Northern parts of China, thereby ensuring a broader representation. Including students from diverse geographical areas would enhance the extrapolation of the present study results to the entire population of university students in China.

Second, this study relied on self-report measures as the sole assessment tool. To overcome this limitation, future research should use diverse measurement methods, such as objective observations, interviews with teachers, parents, or school staff, and the integration of standardized assessments. The incorporation of multiple measurement approaches would enable researchers to conduct a more comprehensive and thorough evaluation of mindfulness in Chinese university students, mitigating potential measurement biases and offering a more accurate depiction.

Third, the cross-sectional design of this study restricts the ability to infer causality from its findings. The survey was conducted in a non-clinical sample of university students. Replicating these findings to a clinical population of university students is highly recommended to comprehensively investigate the relation between mindfulness and psychopathological indicators in university students. Given these limitations, several recommendations emerge for future research. First, efforts should be made to secure a more representative sample of university students from diverse regions of China to enhance the generalizability of survey results. Furthermore, incorporating diverse assessment methods, such as objective observations and standardized assessments, in addition to self-report measures, can provide a more holistic assessment of mindfulness levels and abilities among university students. Longitudinal studies covering multiple academic years should be conducted to capture the dynamic nature of mindfulness levels and abilities among Chinese university students.

Finally, the results show that different components of mindfulness may play different roles in designing interventions. In other words, the eight subscales of the Chinese version of CHIME represent eight different components, which may facilitate accurate intervention design and evaluation. In the future, CHIME can be used as a multifactor evaluation scale to measure intervention effectiveness, avoiding the use of mindfulness total score that can be misleading (Iani et al., 2017). Further, we can explore the mindfulness components that may be enhanced through different practices and design a personalized program of mindfulness skills and abilities for individuals at different stages of practice, allowing them to choose the specific components among the eight mindfulness components they want to cultivate. For example, the practice of breath awareness or body scanning (Hart, 1987) is likely to improve the inner and outer experience awareness or concentration most effectively. The effects of mindfulness training accumulate gradually over time, and CHIME can capture the trajectory of individual mindfulness changes over the course of a mindfulness training intervention, establishing evidence for the effectiveness of the intervention. Cultivating some components of mindfulness may be more challenging than others, suggesting that these skills are acquired gradually. For example, when individuals begin practicing mindfulness, they usually focus on internal and external experiences such as cognition and emotions, and then gradually apply mindfulness skills to view their thoughts and feelings with a nonjudgmental mindset (Lilja et al., 2013). In the later stage, they can explore the interesting changes in terms of the eight components during this dynamic process. Additionally, individual differences in the development of mindfulness skills can be tracked, which can help us understand the relationship between these unique components and the correlation of specific situations such as the type of mindfulness practice, the professional level of meditation, specific psychological disorders, personality types, and the ease of skill learning. The effects of specific components of mindfulness on psychological disorders can be clarified through clinical psychology research, and this may help in treating diseases with a component dysfunction by allowing clinicians to focus on that particular component.

## 7 Conclusion

The Chinese adaptation of the CHIME demonstrates robust psychometric properties and is well-suited for assessing mindfulness in Chinese college students. Moreover, engaging Chinese college students in mindfulness practices may be beneficial for them, contributing to the enhancement of their physical and mental well-being. Mindfulness practice is shown to be effective in regulating emotions, fostering a heightened sense of happiness, and promoting overall wisdom among Chinese college students.

## Data availability statement

The datasets presented in this study can be found in online repositories. The names of the repository/repositories and accession number(s) can be found in the article/supplementary material.

## Ethics statement

The studies involving humans were approved by the Ethics Committee for Psychological Research of the Central China Normal University. The studies were conducted in accordance with the local legislation and institutional requirements. Written informed consent for participation in this study was provided by the participants’ legal guardians/next of kin. Written informed consent was obtained from the individual(s), and minor(s)’ legal guardian/next of kin, for the publication of any potentially identifiable images or data included in this article.

## Author contributions

DZ: Conceptualization, Data curation, Formal analysis, Funding acquisition, Investigation, Methodology, Project administration, Resources, Software, Writing – original draft, Writing – review & editing. JS: Data curation, Formal analysis, Funding acquisition, Methodology, Project administration, Resources, Writing – review & editing. HM: Supervision, Validation, Visualization, Writing – review & editing.
